# Health seeking behaviors and childcare patterns in an informal settlement of Nairobi, Kenya: A cross-sectional study

**DOI:** 10.1371/journal.pgph.0000738

**Published:** 2022-07-14

**Authors:** Derrick Ssewanyana, Linlin Zhang, Marie-Claude Martin, Kerrie Proulx, Tina Malti, Amina Abubakar, Vibian Angwenyi, Margaret Kabue, Joyce Marangu, Rachel Odhiambo, Eunice Njoroge, Eunice Ombech, Mercy Moraa Mokaya, Emmanuel Kepha Obulemire, Greg Moran, Kofi Marfo, Stephen Lye

**Affiliations:** 1 Alliance for Human Development, Lunenfeld-Tanenbaum Research Institute, Toronto, ON, Canada; 2 Key Laboratory of Learning and Cognition, School of Psychology, Capital Normal University, Beijing, China; 3 Centre for Child Development, Mental Health, and Policy, University of Toronto, Mississauga, Canada; 4 Institute for Human Development, Aga Khan University, Nairobi, Kenya; 5 Neuroassement group, KEMRI/Wellcome Trust Research Programme, Centre for Geographic Medicine Research, Kilifi, Kenya; 6 Department of Psychology, Western University, London, ON, Canada; MCRI, University of Melbourne, Royal Children’s Hospital Melbourne, AUSTRALIA

## Abstract

Children in urban informal settlements experience high risks for poor health and development. Understanding health seeking behaviors and childcare patterns of caregivers in urban informal settlements is important for devising effective interventions. This study describes household characteristics and aspects of nurturing care among caregivers of children aged 0–2 years in Dagoretti informal settlement, Nairobi-Kenya. In this cross sectional study, data were collected on household socio-demographic characteristics, antenatal care and child delivery practices, infant and young child feeding practices, activities that promote play, learning and school readiness, and on childcare and protection practices. Descriptive statistics of proportions and means were used to summarize the data. The study covers a total of 458 Kenyan and 118 immigrant households. Caregivers from immigrant households, with low education and from the younger age-group (less than 20 years) were vulnerable to sub-optimal caregiving and health seeking practices, including relatively lower rates of age-appropriate breastfeeding and poor dietary diversity. Seventy-five percent of expectant mothers attended less than four antenatal care visits. Households surveyed had limited possession of children’s books (2% with three or more books), limited access to play materials (43% had two or more play materials), and low paternal involvement in child stimulation and early learning activities (14%). One-third and half of the children were left with inadequate care and disciplined using both violent and non-violent methods, respectively. Our findings highlight the urgent need for contextually appropriate and integrated interventions anchored in the WHO’s nurturing care framework. These interventions can benefit from extensive involvement of caregivers, facility and community-based healthcare workers, policy makers, and other relevant stakeholders.

## Introduction

Rapid rates of urbanization have been recorded globally over the past half-century. It is projected that by 2050, about 60% of the population in low-and middle-income countries (LMICs), inclusive of Sub Saharan Africa (SSA), will reside in urban areas or cities [1]. Rapid urbanization has resulted in poor planning and has over-stretched available health, social protection and education resources in many cities, leading to the mushrooming of slums or informal settlements, especially in LMICs [1,2].

Urban informal settlements in LMICs are typically comprised of a variety of marginalized sub-populations, such as low-income and poor urban dwellers, internal migrants from rural areas, internally displaced people and immigrants fleeing conflict and/or disaster in neighboring countries [3,4]. A wide body of literature from urban informal settlements in LMICs confirms that children and women particularly experience heightened vulnerability to poor health and sub-optimal wellbeing [5–8]. Deprivation and exclusion from important health and social services, coupled with a myriad of problems like crime, poor quality housing, poor sanitation, substance abuse, poor schooling conditions, sexual risk behavior, and climate change implications, among others, are commonly reported in urban informal settlements [3,4,6,8,9]. Conversely, data and indicators from most cities are rarely disaggregated in terms of the type of urban dwelling and thus tend to mask the reality of the huge disparities existing within urban areas [4,10]. Hence, the scale and complexity of social and health needs of caregivers and young children in urban informal settlements is often not well understood [3,4,11].

Kenya is among the LMICs in SSA at early stages of rapid urbanization but with a large proportion (60%) of its urban population residing in informal settlements [12]. About 16% of the 494,289 refugees and asylum seekers in Kenya reside in urban areas (especially Nairobi), however, there is also an estimated 18,500 stateless people in the country [13]. Kenya’s maternal health and childhood indicators are sub-optimal amidst the country’s rapid urbanization. The under-5 child mortality rate and maternal mortality ratio in Kenya currently stands at 41 deaths per 1,000 live births and 342 deaths per 100,000 live births respectively [14]. Existing research indicates that the burden could be worse for urban informal settlements [15]. Other studies from Kenya’s informal settlements report poor child health and development outcomes [7,16–18]. For instance, 37% of infants were not fed within the first hour of birth [17], 40% were not exclusively breastfed for the first 6 months, and poor dietary diversity was commonly experienced by young children [16]. Other reported problems include the subjection of children to harsh disciplinary methods, child neglect and inconsistent guardianship, for example in Kibera and Kangemi informal settlements of Nairobi [7,18]. Research is limited on stimulation and early learning activities for young children who reside in urban informal settlements.

The WHO, World Bank and UNICEF Nurturing Care Framework advocates for improved multi-sectoral coordination, especially during the period from pregnancy to age three [19], when brain development is rapid [20], and children are most susceptible to various environmental influences [21]. To reach their potential, children require five components of nurturing care: children’s good health; adequate nutrition; safety and security; responsive caregiving; and opportunities for early learning [19]. While a strong case has been made for the urgent need to support parents, caregivers and families in providing nurturing care and protection for young children [22], the sheer number of children and families living in sub-optimal urban informal settlements raises the imperative for action and requires the systematic marshalling of research to guide locally relevant and scalable interventions to promote early childhood development.

Currently, information is sparse about practices and conditions around child-rearing in these communities and the needs of families, parents, caregivers and their children. While refugees and asylum seekers are embedded among the Kenyans in urban informal settlements [13], data reported in the majority of studies conducted in Nairobi’s informal settlements are not disaggregated according to immigrant status [10,15,17,23]. Thus, most of the existing literature on Kenya’s urban informal settlements does not optimally provide rich insights on the immigrant sub-population. Our study addresses this gap by inclusion of the immigrant sub-population and disaggregating the data. Besides the isolated reporting on aspects of health and wellbeing, the majority of the studies conducted among Kenyan urban informal settlements have mainly focused on Korogocho and Viwandani [5,15,23–25]. Kenya’s urban informal settlements tend to vary in size, and their needs often vary among and within settlements. Besides, numerous agencies and governmental institutions working in such settings tend to work on specific felt needs, often with little coordination [26]. This study specifically focuses on the child-rearing practices in the informal settlements of Dagoretti sub-county in Nairobi. At the onset of this work, there was hardly any documented research or implementation work in the field of early childhood development from this setting. Unlike Nairobi’s informal settlements of Korogocho and Viwandani which are covered by the Nairobi Urban Health and Demographic Surveillance System since 2003 [27], informal settlements in Dagoretti sub-county have not had routine surveillance thus the living conditions, child rearing practices and intervention needs in these settings are not well understood.

In this survey, we explore the current situation of nurturing care within informal settlements of Dagoretti sub-county in Nairobi-Kenya, as part of formative research work to guide the design of an integrated early child development intervention that operationalizes the nurturing care framework. Specifically, this paper describes the household characteristics and key aspects of nurturing care for children aged 0–2 years.

## Materials and methods

### Study design

A cross-sectional study of households and individuals living in the selected informal settlement communities of Dagoretti sub-county was conducted. The sampling was designed to provide estimates of various indicators about Kenyan and immigrant children of 0–2 years within Dagoretti sub-county. Immigrant households were defined as dwellers whose household head originated from another country besides Kenya. A cluster-based systematic-random sampling method was implemented, whereby a random sample of geographic clusters was drawn and then a random selection of households within each cluster was selected. Community health units were chosen as appropriate sampling frames. In the Kenyan health system, community health units are composed of approximately 1,000 households and are served by approximately 10 community health volunteers (CHVs) [28]. The community units of Kware, Ruthimitu, Centre, Kagira, Matini, Gichungo, Gachui, Githarani, and Nyongara were used as enumeration units and in each, 18 households were to be selected using a systematic-random sampling technique of visiting every 12^th^ household. This strategy was however mainly successful in recruiting an adequate sample of Kenyan households, but the sample of immigrant households was still insufficient. To improve representation of the immigrant sub-population, community refugee leaders were engaged to guide research assistants to purposively identify immigrant households. In consequence, nonprobability sampling methods and expanding the sampling frame to include other community health units within Dagoretti sub-county were employed for the preliminary exploratory study to ensure inclusiveness of immigrant households in informal urban settlements, a population that has been under-researched to date. Due to logistical challenges, coupled with the scarcity of accurate household level data, and the dynamics of community engagement and representation, our decision on the sample size was pragmatic. The sample size depended on as many as possible eligible participants who were recruited through the above sampling approaches.

### Setting and study population

This study was conducted in the informal settlements within Dagoretti sub-county of Nairobi, Kenya. The area is about 12 kilometers from the central business district of Nairobi. By 2019, Dagoretti sub-county accounted for 10% (approximately 434,208 people) of Nairobi’s population [29]. The residents of Dagoretti comprise a diverse background, including rural-to-urban Kenyan migrants and immigrants and asylum seekers from other countries. Access to public health, educational facilities and other social services is constrained within Dagoretti informal settlement. The majority of residents are low income earners who largely depend on the informal sector for a livelihood [30]. In 2019, it was estimated that about 27% of the population of Kawangware County in Dagoretti North constituency lived in informal settlements, however, this estimate may only partly represent the situation of entire Dagoretti sub-county [26].

### Ethics approval and consent to participate

This study was granted ethics approval by the Aga Khan University Institutional Review Board (004-ERC-SSHA-19-EA), and a research permit was granted by the National Commission for Science, Technology & Innovation (NACOSTI/P/19/50782/31710). The study was also reviewed and approved by the Mount Sinai Hospital Research Ethics Board (18-0096-E). All study respondents provided written informed consent.

### Data collection

Data were collected between May and June 2018. Data collection were conducted by 11 trained research assistants (eight females and three males) who initially reviewed all the questionnaire content and underwent practical sessions on interviewing techniques. Each research assistant/interviewer was paired with a CHV or a community refugee leader, who acted as guides and helped create rapport between the research team and interviewees. In the case that the eligible respondent was not present at a household, two extra return visits were made to that household in an effort to improve study participation rate. The survey questionnaire was administered electronically on a tablet using the open data kit (ODK). Upon completion of each interview and verification, data were uploaded to a secure server.

### The survey instrument

The questionnaire items included household demographics, living arrangements, and parenting practices that are known to influence child health, wellbeing and development. Specifically, the contents focused on: antenatal care coverage, childbirth in a health facility, breastfeeding practices, diversity in children’s diet, availability of books and learning materials in the home, parental support for early stimulation and play including fathers’ engagement, and exposure to violent discipline. These items of the survey questionnaire were adapted from the UNICEF Multiple Indicators Cluster Survey (MICS 6) [31] and other health and demographic surveys [32]. A copy of the survey instrument used in this study is included in the *supplementary materials* of this paper for additional information on the structure and content of the instrument.

### Variable definition

This article utilized the following variables from the household survey:

- Socio-demographic and household variables which included *size of the household*, *age* and *sex* of the household members, *highest education level*, *current employment* and *marital status* of the household heads. The *household assets were assessed using* an index indicating the number of items possessed of a standard list of 17 assets, including: bicycle, motorcycle, radio, telephone/mobile, refrigerator, fan, bucket/basin, wooden stool, bed, bed sheets, blankets, mosquito net, chair, table, computer/tablets, access to internet at home and television. *Sources of fuel* (solid fuels, kerosene stove, gas cooker and electric cooker) and *access to sanitation* and *source of drinking water* were assessed. *Living arrangement of children* of 0–2 years was assessed using two separate items asking if the child’s biological mother and biological father reside in the household.
- Health—Maternal and reproductive health variables included the mother’s *antenatal care (ANC) seeking practices* with reference to each child within the age bracket of 0–2 years, *timing of ANC visits*, *number of ANC visits* attended during the pregnancy and *whom their service provider for the ANC during their pregnancy*. We also assessed four aspects on child delivery practices namely: *age at first pregnancy*, *number of live births*, *service provider for assistance during delivery and place of delivery*.
- Nutrition—Infant and young child feeding (IYCF) practices included breastfeeding practices from birth to 24 months and dietary diversity for children from 6–23 months.
- Responsive caregiving and opportunities for early learning—Activities that promote learning and school readiness among children included the *presence of three or more children’s books within the household*, the *proportion of children who have access to two or more types of playthings* (homemade or manufactured toys or household objects), and *the engagement of the child in four or more activities* including reading books or looking at picture books, telling stories, singing songs including lullabies, taking the child outside the home, playing with the child and naming or counting or drawing things for the child by anyone in the households or by mother or by the father.
- Safety and security—Childcare and protection practices for children of age 0–2 years measured two components: 1) *if the child was left alone or in the care of another child who is less than 10 years old for more than an hour in the past week* and 2) *the child discipline methods used by the caregiver or any adult in the household in the previous month* (any form of violent discipline methods, physical aggression, psychological aggression, non-violent discipline, a combination of violent and non-violent discipline).

### Statistical analysis

All the data were analyzed in STATA 15 software package. Social demographic characteristics of age, sex, marital status, education status, religious affiliation, employment, living arrangement and access to sanitation and drinking water across Kenyan and Immigrant households were summarized using descriptive statistics of proportions (%) and means with standard deviation. Bivariate analyses, Chi-square test or Fisher exact test for categorical variable, and T-tests or analysis of variance (ANOVA) were used to test for significance of differences across Kenyan and Immigrant households. We specifically selected one child (of age 0–2 years) per household to minimize data dependency for all subsequent analyses on childhood health, IYCF practices, activities to promote learning and school readiness, and childcare and protection practices. During the selection process of one child per household, for households with more than one child of 0–2 years, the youngest child was selected, and in the case that there were twins or triplets, a child was selected randomly. Analyses on childcare, ANC, IYCF, activities that promote learning and school readiness, and childcare and protection practices were disaggregated by type of household, sex of the child, mother’s age at delivery and maternal education status. A sub-category for missing data was included for variables which had some missing values.

## Results

### Survey population

A total of 458 Kenyan and 118 immigrant households representing 1840 (80%) Kenyans and 607 (20%) immigrants were surveyed. Initially, 674 households had been sampled, however, there was no one at home to contact during the survey among 78 households (12%). Of the eligible 596 households, 3% (20 households) declined to take part in the survey. There were 612 children aged 0–2 years and 80% of them were from Kenyan households.

### Household characteristics

Household characteristics are summarized in **Table 1**. On average, households comprised of four members however, the size of household was significantly larger among the immigrants. A significantly greater proportion of children (below 18 years) and twice as many female-headed households were found among the immigrants compared to Kenyans. The proportion of household heads with primary or lower education level as their highest education was significantly higher among immigrants (40%) than Kenyans (31%). Fifty-seven percent of Kenyan household heads either had paid employment or operated a business, as compared to 41% of the immigrant household heads. More household assets were reported among Kenyan than immigrant households. The use of solid fuels was significantly higher among immigrant households. The greater proportion of Kenyan (80%) as compared to immigrant (67%) children lived with both biological parents. Maternal education attainment was significantly lower for the immigrant children compared to their Kenyan counterparts. About 55% of immigrant mothers versus 37% of Kenyan mothers had primary or lower education level as their highest attained education (**Table 2)**.

**Table 1:** Socio-demographic and household characteristics of study participants.

Characteristics	Type of Household Kenyan N (%)	Immigrants N (%)	P-value
**Size of Household** (mean)	4.03 (±1.3)	5.06 (±2.3)	<0.001
**Age distribution**			0.001
Children (0-17years)	899 (48.9)	347 (57.2)	
Adults (18 years and above)	916 (49.8)	256 (42.2)	
Missing/DK	23 (1.3)	4 (0.7)	
**Sex of household head**			<0.001
Male	387 (84.5)	80 (67.8)	
Female	71 (15.5)	38 (32.2)	
**Marital status of household head**			0.06
Never married	24 (5.2)	11 (9.3)	
Currently married	393 (85.8)	95 (80.5)	
Widow/Widower	11 (2.4)	7 (5.9)	
Divorced/Separated	30 (6.6)	5 (4.2)	
**Education level of household head**			<0.001
Never attended any level	10 (2.2)	8 (6.8)	
Primary level	130 (28.4)	39 (33.0)	
Secondary level	229 (50.0)	31 (26.3)	
Above Secondary	67 (14.6)	34 (28.8)	
Missing/DK	22 (4.8)	6 (5.1)	
**Employment of household head**			<0.001
Paid employee	I68 (36.7)	19 (16.1)	
Owns business or employer	93 (20.3)	29 (24.6)	
Owns farm or keeps livestock	0 (0.0)	1 (0.9)	
Casual labour	166 (36.2)	39 (33.1)	
Unpaid family worker or housewife	16 (3.5)	22 (18.6)	
Apprentice or other forms	3 (0.7)	3 (2.5)	
Not applicable	10 (2.2)	4 (3.4)	
Missing/DK	2 (0.4)	1 (0.9)	
Assets in the household (mean)	9.5 (±1.9)	8.4 (±2.1)	<0.001
**Sources of fuel in the household**			<0.001
Solid fuels	32 (7.0)	20 (16.9)	
Kerosene stove	206 (45.0)	63 (53.4)	
Gas cooker	219 (47.8)	35 (29.7)	
Electric cooker	1(0.2)	0 (0.0)	
**Access to sanitation**			0.908
Improved sanitation facilities	286 (79.7)	172 (79.3)	
Unimproved sanitation facilities	73 (20.3)	45 (20.7)	
**Source of drinking water**			0.276
Protected water source	330 (72.1)	79 (66.9)	
Unprotected water source	128 (27.9)	39 (33.1)	

DK: Don’t know; ±: Standard deviation; *P-values for continuous variables are a product of T-test or ANOVA and for binary/categorical variables are from Chi-square or Fisher’s exact test*.

**Table 2:** Selected background characteristics of children of age two years and under.

Characteristics	All under-2 children (n = 612)		P-value	Selected Under-2 children (1 child per household) n = 575		P-value
	Kenyan N (%)	Immigrants N (%)		Kenyan N (%)	Immigrants N (%)	
**Sex of the child**			0.729			0.647
Male	237 (48.7)	63 (50.4)		224 (48.9)	60 (51.3)	
Female	250 (51.3)	62 (49.6)		234 (51.1)	57 (48.7)	
Child age (months)			0.566			0.383
0–5	98 (20.1)	22 (17.6)		98 (21.4)	22 (18.8)	
6–11	113 (23.2)	23 (18.4)		112 (24.4)	23 (19.7)	
12–23	212 (43.5)	58 (46.4)		195 (42.6)	52 (44.4)	
24–35	61 (12.5)	21 (16.8)		52 (11.4)	19 (16.2)	
Missing/DK	3 (0.6)	1 (0.8)		1 (0.2)	1 (0.9)	
**Living arrangement**			0.003			0.001
Lives with both biological parents	389 (80.1)	84 (67.2)		370 (80.8)	79 (67.5)	
Lives with only one of the biological parents	74 (15.2)	25 (20.0)		70 (15.3)	23 (19.7)	
Lives with none of the biological parents	1 (0.2)	0 (0.0)		0 (0.0)	0 (0.0)	
Others	22 (4.5)	16 (12.8)		18 (3.9)	15 (12.8)	
**Maternal education**			<0.001			<0.001
Never attended any level	7 (1.4)	13 (10.4)		6 (1.3)	13 (11.1)	
Primary level	173 (35.5)	56 (44.8)		161 (35.1)	51 (43.6)	
Secondary level	229 (47.0)	41 (32.8)		217 (47.4)	38 (32.5)	
Above Secondary	68 (14.0)	11 (8.8)		65 (14.2)	11 (9.4)	
Missing/DK	10 (2.1)	4 (3.2)		9 (2.0)	4 (3.4)	

DK: Don’t know; Others (included those who had some missing data on one of the parent); *P-values for binary/categorical variables are from Chi-square or Fisher’s exact test*.

### Maternal and reproductive health

The patterns of antenatal care (ANC) seeking practices of mothers during pregnancy (index child) are summarized in **Table 3**. The proportions of mothers who attended 4 or more ANC visits (75%) and who received ANC from a trained healthcare worker (98%) were high. The number of livebirths (parity) was significantly higher among immigrant mothers, older women and mothers with lower education attainment. There were no significant differences in timing of ANC visits, number of ANC visits and service provider for ANC across household types, maternal age groups and maternal education levels. The larger proportion of women had their first pregnancy after 18 years of age regardless of their education attainment (**Table 4**).

**Table 3:** Antenatal care seeking practices of mothers during their pregnancy for the previous birth.

Characteristics	Timing of ANC visits (n = 575)				Number of ANC visits (n = 575)				Service provider for ANC (n = 575)			
	Before 3 months pregnant	After 3 months pregnant	Don’t remember	P-value	1–3 visits	4+ visits	Don’t remember	P-value	Healthcare worker*	Traditional birth attendant	No one	P-value
**Type of household**				0.354				0.059				0.146
Kenyan	118 (25.8)	331 (72.3)	9 (1.9)		117 (25.6)	328 (71.6)	13 (2.8)		452 (98.7)	1 (0.2)	5 (1.1)	
Immigrant	38 (32.5)	77 (65.8)	2 (1.7)		19 (16.2)	92 (78.6)	6 (5.1)		113 (96.6)	1 (0.8)	3 (2.6)	
**Mother’s age at birth**				0.460				0.525				0.353
Less than 20	4 (20.0)	16 (80.0)	0 (0.0)		5 (25.0)	14 (70.0)	1 (5.0)		20 (100.0)	0 (0.0)	0 (0.0)	
20–34 years	126 (28.1)	313 (69.9)	9 (2.0)		101 (22.5)	331 (73.9)	16 (3.6)		442 (98.7)	1 (0.2)	5 (1.1)	
35 and above	25 (25.3)	73 (73.7)	1 (1.0)		26 (26.3)	71 (71.7)	2 (2.0)		95 (96.0)	1 (1.0)	3 (3.0)	
DK/Missing	1 (12.5)	6 (75.0)	1 (12.5)		4 (50.0)	4 (50.0)	0 (0.0)		8 (100.0)	0 (0.0)	0 (0.0)	
**Mother’s education level**				0.392				0.454				0.368
Never attended any level	4 (21.0)	14 (73.7)	1 (5.3)		3 (15.8)	15 (78.9)	1 (5.3)		18 (94.7)	0 (0.0)	1 (5.3)	
Nursery & Primary level	56 (26.4)	150 (70.8)	6 (2.8)		55 (25.9)	149 (70.3)	8 (3.8)		206 (97.2)	2 (0.9)	4 (1.9)	
Secondary level	71 (27.8)	182 (71.4)	2 (0.8)		61 (23.9)	185 (72.6)	9 (3.5)		253 (99.2)	0 (0.0)	2 (0.8)	
Above Secondary	21 (27.6)	54 (71.1)	1 (1.3)		12 (15.8)	63 (82.9)	1 (1.3)		75 (98.7)	0 (0.0)	1 (1.3)	
Missing/DK	4 (30.8)	8 (61.5)	1 (7.7)		5 (38.5)	8 (61.5)	0 (0.0)		13 (100.0)	0 (0.0)	0 (0.0)	

Healthcare worker*: A doctor or nurse or midwife; ANC: Antenatal Care; DK: Don’t know; *P-values for binary/categorical variables are from Chi-square or Fisher’s exact test*.

**Table 4:** Child delivery practices among mothers of children aged 0–2 years.

Characteristics	Age at first pregnancy			Live births		Assistance during delivery						Place of delivery	
	Less than 18	After 18	P-value	Mean (SD)	P-value	Doctor, nurse, midwife	Traditional birth attendant	Community health worker	Relative or friend	No one	P value	Health facility	P-value
**Type of household**			0.132		0.0003						0.375		0.710
Kenyan	51 (11.1)	407 (88.9)		2.1 (±1.3)		440 (96.1)	8 (1.8)	0 (0.0)	4 (0.9)	6 (1.3)		436 (95.2)	
Immigrant	19 (16.2)	98 (83.8)		2.9 (±2.0)		114 (97.3)	0 (0.0)	1 (0.9)	1 (0.9)	1 (0.9)		114 (97.4)	
**Mother’s age at birth**			<0.001		<0.001						0.950		0.860
Less than 20	10 (50.0)	10 (50.0)		1.1 (±0.3)		20 (100.0)	0 (0.0)	0 (0.0)	0 (0.0)	0 (0.0)		20 (100.0)	
20–34 years	43 (9.6)	405 (90.4)		2.2 (±1.4)		431 (96.2)	7 (1.6)	1 (0.2)	4 (0.9)	5 (1.1)		427 (95.3)	
35 and above	13 (13.1)	86 (86.9)		3.3 (±1.9)		95 (96.0)	1 (1.0)	0 (0.0)	1 (1.0)	2 (2.0)		95 (95.9)	
DK/Missing	4 (50.0)	4 (50.0)		1.9 (±1.1)		8 (100.0)	0 (0.0)	0 (0.0)	0 (0.0)	0 (0.0)		8 (100.0)	
**Mother’s education level**			<0.001		<0.001						0.514		0.328
Never attended any level	8 (42.1)	11 (57.9)		3.3 (±2.3)		18 (94.7)	0 (0.0)	1 (5.3)	0 (0.0)	0 (0.0)		18 (94.7)	
Primary level	32 (15.1)	180 (84.9)		2.7 (±1.7)		200 (94.3)	5 (2.4)	0 (0.0)	3 (1.4)	4 (1.9)		197 (92.2)	
Secondary level	21 (8.2)	234 (91.8)		2.1 (±1.3)		247 (96.9)	3 (1.2)	0 (0.0)	2 (0.8)	3 (1.2)		246 (96.5)	
Above Secondary	3 (3.9)	73 (96.1)		1.7 (±0.9)		76 (100)	0 (0.0)	0 (0.0)	0 (0.0)	0 (0.0)		76 (100.0)	
Missing/DK	6 (46.2)	7 (53.8)		1.9 (±1.4)		13 (100)	0 (0.0)	0 (0.0)	0 (0.0)	0 (0.0)		13 (100.0)	

±: Standard deviation; DK: Don’t know; *P-values for binary/categorical variables are from Chi-square or Fisher’s exact test*.

### Infant and young child feeding (IYCF) practices

The majority (95%) of children aged 0–2 years had been breastfed at some time, with the greater proportion being among Kenyan than immigrant children. There were however no differences by sex of the child (**Table 5**). The proportion of children who had been breastfed was significantly lowest among mothers below 20 years (at the time of their child’s birth). Overall, 68% of the children were initiated to breastfeeding within the first hour after birth, with similar results across Kenyan and immigrant children. Findings on attainment of dietary diversity indicated that a significantly higher proportion of Kenyan (39%) than immigrant (21%) children aged 6–23 months received a diverse diet. Age-appropriate breastfeeding, defined as predominant breastfeeding of children aged 0–5 months and continued breastfeeding complemented with solid, semi-solid or soft foods for children 6–23 months, was least reported among children of the youngest mothers (below 20 years).

**Table 5:** A summary of Infant and Young child feeding practices of children ages 0–2 years.

Characteristics	Ever breastfed		Initiation of breastfeeding			Age-appropriate breastfeeding				Dietary diversity	
	Yes	p-value	Within 1 hour	Within 24 hours	p-value	**0–5** (n = 120)	**6–11** (n = 135)	**12–23** (n = 247)	p-value	**6–23** (n = 382)	p-value
**Type of household**		0.042			0.177				0.065		0.003
Kenyan	446 (97.4)		310 (67.7)	119 (26.0)		71 (72.5)	101 (90.2)	151 (77.4)		121 (39.4)	
Immigrant	109 (93.2)		80 (68.4)	24 (20.5)		15 (68.2)	20 (86.9)	34 (65.4)		16 (21.3)	
**Sex of child**		0.496			0.056				0.220		0.893
Male	276 (97.2)		179 (63.0)	83 (29.2)		41 (70.7)	63 (90.0)	91 (70.5)		72 (36.2)	
Female	279 (95.9)		211 (72.5)	60 (20.6)		45 (72.6)	58 (89.2)	94 (79.7)		65 (35.5)	
**Mother’s age at birth**		0.019			0.125				0.026		0.286
Less than 20	17 (85.0)		11 (55.0)	5 (25.0)		2 (50.0)	2 (100.0)	5 (62.5)		3 (30.0)	
20–34 years	436 (97.3)		305 (68.1)	113 (25.2)		66 (72.5)	97 (92.4)	156 (78.4)		107 (35.2)	
35 and above	95 (95.9)		69 (69.7)	24 (24.2)		18 (72.0)	18 (75.0)	24 (64.9)		22 (36.1)	
DK/Missing	7 (87.5)		5 (62.5)	1 (12.5)		-	4 (100.0)	0 (0.0)		5 (71.4)	
**Mother’s education level**		0.222			-				0.877		0.064
Never attended any level	17 (89.5)		13 (68.4)	2 (10.5)		0 (0.0)	5 (71.4)	7 (77.8)		1 (6.3)	
Nursery & Primary level	204 (96.2)		155 (73.11)	43 (20.3)		33 (73.3)	47 (85.5)	63 (74.1)		50 (35.7)	
Secondary level	247 (96.9)		171 (67.1)	68 (26.7)		39 (69.4)	55 (94.8)	84 (77.1)		60 (35.9)	
Above Secondary	75 (98.7)		42 (55.3)	28 (36.8)		12 (80.0)	10 (90.9)	28 (73.7)		21 (42.9)	
Missing/DK	12 (92.3)		9 (69.2)	2 (15.4)		2 (100.0)	4 (100.0)	3 (50.0)		5 (50.0)	

P-values for binary/categorical variables are from Chi-square or Fisher’s exact test.

### Activities that promote learning and school readiness among children

In total, 70% of Kenyan respondents and 54% of immigrant respondents reported that a household member engaged the child in at least four or more activities that promote learning and school readiness during the three days prior to the survey (see **Table 6**). A higher proportion of males than female children were engaged in four or more such activities. Noteworthy, very few children (15% Kenyan and 13% immigrants) were engaged by their fathers in four or more activities that promote learning. Fathers whose spouses had better education status were found to have a significantly greater level of engagement with their children in these activities. Possession of children’s books or picture books was extremely low as only 2% of Kenyan and none of the immigrant households possessed three or more children’s books or picture books. Notably, only households with mothers of higher education level (secondary level and above) were in possession of these children’s books or picture books. Below one-half of the Kenyan (48%) and immigrants (37%) children had access to two or more types of playthings. There was a significantly greater proportion of children that had access to two or more types of playthings among mothers with high education attainment.

**Table 6:** Engagement in activities that promote learning and school readiness among children ages 0–2 years.

Characteristics	Mean number of activities and % of children with whom:									Children’s books in the household		% of children who play with	
	anyone has engaged in four or more activities	Mean number of activities with anyone	P-value*	fathers have engaged in four or more activities	Mean number of activities with fathers	P-value*	mothers have engaged in four or more activities	Mean number of activities with mothers	P-value*	3 or more children’s books	P-value	2 or more types of playthings	P-value
**Type of household**			0.001			0.503			0.106		0.370		0.026
Kenyan	322 (70.3)	4.2 (±1.4)		70 (15.3)	1.2 (±1.7)		238 (51.9)	3.5 (±1.6)		8 (1.8)		221 (48.3)	
Immigrant	63 (53.8)	3.8 (±1.7)		15 (12.8)	1.2 (±1.8)		51 (43.6)	3.2 (±1.6)		0 (0.0)		43 (36.8)	
**Sex of child**			0.054			0.099			0.007		0.286		0.285
Male	201 (70.7)	4.2 (±1.4)		49 (17.2)	1.3 (±1.8)		159 (55.9)	3.6 (±1.6)		2 (0.7)		124 (43.7)	
Female	184 (63.2)	4.0 (±1.5)		36 (12.4)	1.2 (±1.7)		130 (44.7)	3.2 (±1.6)		6 (2.1)		140 (48.1)	
**Mother’s age at birth**			0.237			0.354			0.587		0.182		0.941
Less than 20	13 (65.0)	4.1 (±1.5)		3 (15.0)	1.3 (±1.6)		10 (50.0)	3.8 (±1.5)		0 (0.0)		9 (45.0)	
20–34 years	299 (66.7)	4.1 (±1.4)		72 (16.1)	1.4 (±1.8)		230 (51.3)	3.5 (±1.5)		6 (1.3)		208 (46.4)	
35 and above	65 (65.7)	4.0 (±1.7)		10 (10.1)	0.8 (±1.6)		44 (44.4)	3.1 (±1.8)		1 (1.0)		43 (43.4)	
DK/Missing	8 (100.0)	5.5 (±0.7)		0 (0.0)	0.0 (0.0)		5 (62.5)	4.2 (±1.7)		1 (12.5)		4 (50.0)	
**Mother’s education level**			0.388			0.053			0.266		0.006		P<0.001
Never attended any level	10 (52.6)	3.70 (±1.6)		2 (10.5)	0.9 (±1.6)		6 (31.6)	2.5 (±1.5)		0 (0.0)		8 (42.1)	
Nursery & Primary level	138 (65.1)	4.0 (±1.5)		23 (10.8)	1.0 (±1.5)		100 (47.2)	3.3 (±1.6)		0 (0.0)		78 (36.8)	
Secondary level	173 (67.8)	4.2 (±1.3)		40 (15.7)	1.3 (±1.8)		133 (52.2)	3.6 (±1.5)		3 (1.2)		123 (48.2)	
Above Secondary	53 (69.7)	4.2 (±1.6)		19 (25.0)	1.9 (±1.9)		42 (55.3)	3.5 (±1.8)		4 (5.26)		51 (67.1)	
Missing/DK	11 (84.6)	4.8 (±1.6)		1 (7.7)	0.4 (±1.4)		8 (61.5)	3.2 (±1.6)		1 (7.7)		4 (30.8)	

Types of play things are in form of: Homemade toys, toys from a shop/manufactured toys and household objects/objects found outside. ±: Standard deviation; P-value*: P-value for Chi-square test of differences in engagement in 4 or more activities; *P-values for continuous variables are a product of T-test or ANOVA and for binary/categorical variables are from Chi-square or Fisher’s exact test*.

### Child protection practices for children

About one-third (33% of Kenyans and 37% of immigrants) of children of both sex aged 0–2 years were left with inadequate care in the week preceding the survey, that is, either left alone for more than one hour or left in the care of another child under age 10 years (**see Table 7**). Compared to younger mothers, the older mothers reported a significantly greater proportion of children left with inadequate care. The majority (73%) of the children had experienced some form of violent discipline in the past month. About 68% of the children had experienced psychological aggression (e.g. being yelled, screamed or shouted at) whereas 57% of them experienced physical aggression (e.g. being spanked or hit or slapped). Only 4% of the children had experienced only non-violent discipline whereas 53% had experienced a combination of violent and non-violent discipline in the past month. There were no significant differences in disciplinary practices across household types, maternal age groups and maternal education levels.

**Table 7:** Childcare and protection practices for children ages 0–2 years.

Characteristics	Inadequate care within the past week				Child disciplining methods during the previous month				
	Left alone for more than an hour in the past week n(%)	Left in the care of another child younger than 10 years of age n(%)	Left with inadequate care in the past week n(%)	*P-value	Experienced any form of violent discipline n(%)	Experienced physical aggression n(%)	Experienced Psychological aggression n (%)	Experienced only non-violent discipline n(%)	Experienced a combination of violent and non-violent discipline (n%)
**Type of household**				0.388					
Kenyan	108 (23.6)	96 (21.0)	149 (32.5)		346 (75.5)	281 (61.4)	321 (70.1)	18 (3.9)	242 (52.8)
Immigrant	31 (26.5)	28 (23.9)	43 (36.8)		82 (70.1)	61 (52.1)	78 (66.7)	5 (4.3)	63 (53.8)
**Sex of child**				0.498					
Male	65 (22.9)	58 (20.4)	91 (32.0)		215 (75.7)	173 (60.9)	206 (72.5)	9 (3.2)	150 (52.8)
Female	74 (25.4)	66 (22.7)	101 (34.7)		213 (73.2)	169 (58.1)	193 (66.3)	14 (4.8)	155 (53.3)
**Mother’s age at birth**				0.005					
Less than 20	3 (15.0)	3 (15.0)	3 (15.0)		15 (75.0)	12 (60.0)	15 (75.0)	0 (0.0)	12 (60.0)
20–34 years	105 (23.4)	89 (19.9)	142 (31.7)		338 (75.5)	267 (59.6)	315 (70.3)	18 (4.0)	241 (53.8)
35 and above	28 (28.3)	27 (27.3)	41 (41.4)		69 (69.7)	58 (58.6)	63 (63.6)	5 (5.1)	48 (48.5)
DK/Missing	3 (37.5)	5 (62.5)	6 (75.0)		6 (75.0)	5 (62.5)	6 (75.0)	0 (0.0)	4 (50.0)
**Mother’s education level**				0.207					
Never attended any level	2 (10.5)	2 (10.5)	4 (21.0)		12 (63.2)	11 (57.9)	12 (63.2)	3 (15.8)	10 (52.6)
Nursery & Primary level	53 (25.0)	52 (24.5)	73 (34.4)		152 (71.7)	118 (55.7)	145 (68.4)	9 (4.3)	108 (50.9)
Secondary level	59 (23.1)	52 (20.4)	83 (32.5)		194 (76.1)	157 (61.6)	176 (69.0)	9 (3.5)	135 (52.9)
Above Secondary	20 (26.3)	12 (15.8)	24 (31.6)		61 (80.3)	50 (65.8)	57 (75.0)	2 (2.6)	46 (60.5)
Missing/DK	5 (38.5)	6 (46.2)	8 (61.5)		9 (69.2)	6 (46.2)	9 (69.2)	0 (0.0)	6 (46.2)

*P-values for continuous variables are a product of T-test or ANOVA and for binary/categorical variables are from Chi-square or Fisher’s exact test;* *P-value: p-value in relation to the combined variable on “left with inadequate care in the past week”; None of the child disciplining methods had statistical significance.

## Discussion

The findings highlight that immigrant status (i.e. being an immigrant), low education attainment by mothers, and young age of caregivers seem likely to be associated with poorer maternal health seeking behaviors and child care patterns. Although this is a cross sectional study where causation cannot be inferred, we propose that these factors may increase vulnerability of caregivers and their children to ill health, sub-optimal child development, and poor livelihood in Dagoretti’s informal settlements. Similar to our findings, other studies conducted in Nairobi’s informal settlements by Fosto and colleagues [33], Arnold et al. [34], and Faye et al. [35], have linked low maternal education, poverty (especially food and assets poverty), lower maternal age (e.g. below 18 years), and the experience of unique barriers by migrants in accessing healthcare to increased vulnerability to sub-optimal health, and development among urban poor infants and children. The lack of legal documentation among some urban immigrants may further increase the barriers to employment, healthcare and other crucial services [36,37]. The implications of these factors, if left unaddressed, are that socio-economic and health disparities are likely to widen between Kenyan and immigrant families within the informal settlement but also in a broader perspective, between the slum dwellers and the rest of the urban population.

Our findings show that women in Dagoretti’s informal settlement have close to universal access to ANC services. This finding is consistent with Kenya’s national level estimates of 96% access to ANC by a skilled health provider [32], and by reports from two informal settlements of Korogocho and Viwandani in Nairobi city [10]. We found, however, that such universal access to ANC did not guarantee optimal ANC seeking behavior. That is, very few expectant women (about 26–33%) sought timely ANC within their first trimester and one-quarter of them did not attend at least four ANC visits as recommended by the World Health Organization [38]. Besides, some studies from Kenyan informal settlements indicate that, whereas access to some maternal health services may seem universal, the majority of these service providers lack the requisite skills, are under staffed, and lack various essential services [10,23]. Although our findings for both timing and frequency of ANC visits are very similar to the overall estimates within Nairobi city (based on Kenya Demographic and Health Survey) [32], they support the need for improved community mobilization and health promotion to sensitize and empower expectant women and their partners to observe timely and appropriate frequency of ANC.

Similar to our findings on sub-optimal child feeding practices, especially poor exclusive breastfeeding and low dietary diversity, studies from other informal settlements of Nairobi have reported poor child nutrition outcomes and recommended for age-appropriate interventions tackling the underlying factors [16,17,24]. Some feasible interventions may include home-based nutrition education and counseling during pregnancy through trained CHVs, training programmes for health workers on proper counseling on IYCF, empowerment of mothers through support groups, and demonstration on IYCF technics [25,39].

Our findings indicate limited engagement of children in activities that promote learning and school readiness coupled with low access to a variety of play materials, especially for immigrant children and children of caregivers having low education attainment. Currently, research on child play, stimulation and early learning activities for young children below 3 years and living in urban informal settlements is scanty within the Kenyan context. Data from non-urban informal settings of Kenya, such as the 2013–14 multiple indicator surveys conducted in Bungoma [40], and Kakamega [41] counties, also indicated poor outcomes, but they were better than those from our current study. For instance, the availability of three or more children’s books in households (4%), two or more child play materials (54–69%), and child engagement in four or more activities (63–74%) were higher in both Bungoma [40] and Kakamega [41], when compared to our findings in Dagoretti. Besides, the extremely low involvement of fathers in caregiving practices in Dagoretti was a concern. An extensive body of evidence on potential barriers for male caregivers’ involvement in early childhood caregiving includes factors such as negative socio-cultural stereotypes and beliefs, inadequate knowledge on benefits of early childhood stimulation, fathers who live away from their children, and maternal reluctance to involve the fathers [42,43]. There is thus an urgent need for contextualized parenting support for fathers in Dagoretti to enhance the quality of father-child interaction skills and their involvement with children. Also, the need to tailor interventions which stimulate early learning, language and cognitive development is timely for caregivers and their families in Dagoretti.

Our findings on child protection practices indicate that both psychological and physical forms of violent disciplinary practices are common and that for the most part, caregivers employed a combination of violent and non-violent disciplinary practices. Our findings on high prevalence of violent disciplinary practices are corroborated by data from the 2013–14 MICS in Kakamega and Bungoma counties where an estimated 82% of children age 1–14 years had experienced psychological aggression or physical punishment during the last one month [40,41]. This may suggest that child protection is still highly problematic and this issue cuts across Kenyan communities. Research findings from LMICs indicate that caregivers who believe that proper child rearing requires physical punishment tend to administer violent discipline but paradoxically, a large proportion of those who felt that physical punishment was unnecessary still practiced it [44]. Although we anticipate that the experience of daily life stressors may partly explicate the existing poor child rearing practices, there is need for further investigation to better understand the factors behind the use of violent discipline within this setting. It is crucial to reinforce existing positive methods of child rearing and to train parents on how to positively interact with their children.

The findings from this study have been instrumental in guiding a community engagement process [45], and other stages of an ongoing integrated early child development (ECD) intervention that operationalizes the nurturing care framework [46]. For community engagement, the findings from this study were shared and discussed with various Ministry of Health stakeholders, such as the Dagoretti sub-County Health Management Team and other actors, such as community-based organizations and service providers in Dagoretti. The sharing of findings helped in facilitating dialogue on priority areas for interventions, created buy-in, and was useful for mapping potential community resources to consider during the planning and implementation of the ECD intervention [45]. Highlights from this work, such as child-rearing practices, father involvement, child feeding patterns, maternal stressors and social determinants of health, were crucial in designing contextually relevant training modules and improving experiential learning during training of community health volunteers who are currently serving as the integrated ECD intervention delivery agents. As an important aspect of the integrated ECD intervention design, the findings from this study were fundamental in the identification and prioritization of the following specific topics as key components of the ongoing integrated ECD package: cognitively stimulating parent-child interactions and play; maternal, infant and young child nutrition; paternal involvement in child caregiving, social cohesion; maternal mental wellbeing, and economic empowerment [46].

Some of the study limitations include the use of self-reported measures which can be subject to recall-bias and self-desirability bias. Nonetheless, self-reported measures on population health and childhood development are conventionally utilized and proven to yield valid data [47]. We did not perform multi-variable analysis to confirm the associations across variables and this should be done in future studies, preferably with larger sample size. Besides, our sample of Kenyan households was much larger than that of immigrant household which could have presented some limitations in drawing significant comparisons. A potential limitation of this study is that we did not include childcare outside of the home, for example children within the foster care system and children who receive daycare at child-care centers. This is a potential area for future research. Another study limitation is that due to the large number of variables reported, there might have been a risk of type 1 error and thus the significance of differences across groups should be interpreted with some level of caution. Besides, this being a cross sectional study, the direction of the associations and causation could not be inferred from our results. Nevertheless, the immigrant sub-population is much less than that of Kenyans within this setting. Besides, we utilized mixed sampling techniques to improve the inclusiveness of immigrant sup-populations in the study findings. Thus, the results from this exploratory study should be interpreted with caution as they are not intended to be generalizable to other urban informal settlements or representative of the wider populations. Lastly, the selection of one child (of age 0–2 years) per household to minimize data dependency may have introduced some aspect of misrepresentation of the general status of outcomes for the other children in the same age-bracket within the households that had more than one child fitting this age criteria.

## Conclusions

Taken together, many families with children aged 0–2 years in Dagoretti informal settlement experience multi-faceted sources of risk for poor health and livelihood. Most notable among the vulnerable families are immigrant households, households with younger caregivers that is, below 20 years of age and caregivers with low maternal education. Furthermore, infant and young child feeding practices (exclusive breastfeeding and dietary diversity); child stimulation (play, early learning and paternal involvement); and childcare and protection (especially disciplinary practice and adequate care) were key areas with major inadequacies. We used the findings of this work to inform our contextually relevant community engagement process and in designing an integrated intervention package on early child development in the urban informal settlements in Dagoretti. We recommend that intervention studies should be guided by formative research especially in dynamic contexts like urban informal settlements where local and migrant populations co-exist.

## Supporting information

S1 Checklist A STROBE checklist of this cross-sectional study. (DOCX)

S1 Questionnaire The English version of the questionnaire administered in the cross-sectional study. (PDF)
