# Supplementary material for: Health seeking behaviors and childcare patterns in an informal settlement of Nairobi, Kenya: A cross-sectional study
Source: PLOS Glob Public Health. 2022 Jul 14;2(7):e0000738. doi: 10.1371/journal.pgph.0000738 (PMC10021912; doi:10.1371/journal.pgph.0000738)
Supplement: S1 Questionnaire — (PDF) [file pgph.0000738.s002.pdf]

FILE 2: THE ENGLISH VERSION OF THE QUESTIONNAIRE ADMINISTERED IN THE CROSS-SECTIONAL STUDY

CDMC HOUSEHOLD SURVEY

February 22, 2018

Instructions: Hello, my name is (*your name*). We are from (*your affiliation*). We are planning a project to help young children in this community. We are conducting a survey to understand children's and their families' needs so we can provide better service. I would like to talk to somebody who can answer on behalf of the household. I would like also to speak with anyone who cares for children less than 2 years old. The questions take about (*number*) minutes to answer. We would greatly appreciate your co-operation in this research. You may decide not to be questioned if you would prefer. But we will be happy if you agree because your experience and your suggestions are very important. The information that you provide us with will be kept confidential, and is for research purposes only. Your name will not appear in any report that comes out of this study.

**Interview Details**	
**Country**	**Kenya**
**City**	**Nairobi**
**District**	**Kawangware**
**Village**	
**Cluster**	
**Household**	

**Interview Date**								**Time at Start:**				**Time at Finish:**			
**d**	**d**	**m**	**m**	**y**	**y**	**y**	**y**	**h**	**h**	**m**	**m**	**h**	**h**	**m**	**m**

Main Interviewee		Consent Given	
Name	ID	YES	1
		NO	2

**Outcome of first visit**	**Code**
Completed	01
No household member at home or no competent respondent at home at time of visit	02
Entire household absent for extended period of time	03
Postponed	04
Refused	05
Dwelling vacant or address not a dwelling	06
Dwelling destroyed	07
Dwelling not found	08
Other (specify)______________________________________	97

**Outcome of second visit**	**Code**
Completed	01
No household member at home or no competent respondent at home at time of visit	02
Refused	05
Other (specify)______________________________________	97

Census Code		
GPS Co-ordinates	S	
	E	

**Interviewer Details**	**Name:**	**ID Code:**	**Date: (ddmmyyy)**								**Signature**
**Interviewer**											
**Checked by**											

**Data entered by**											

PANEL 1. HOUSEHOLD DEMOGRAPHY AND INFRASTRUCTURE

In this panel, we would like to talk to the head of the household or an adult who knows about the household. We would like to know about each person who uses this household as their main place of residence, even if they may be away for long periods of time to work, receive education, or visit relatives. Please include any children who are fostered into the household.

1.1. List of Household Members

In this section, I am going to ask about some basic information of each household member.

**HL0**.<br>*ID*	**HL1**.<br>First, please tell me the name of each person who usually lives here, starting with the head of the household.<br><br>*Probe for additional household members.*	**HL2**.<br>What is the relationship of (***name***) to (***name of the head of household***)?	**HL3**.<br>Is (***name***) male or female?<br><br>1 male<br>2 female	**HL4**.<br>What is (**name**)'s date of birth?<br><br>Month:<br>98 Don't know<br>Year:<br>9998 Don't know	**HL5**.<br>How old is (***name***)?<br><br>*Record in completed years.*	**HL6**.<br>What is (**name**)'s marital status?<br><br>1 never married<br>2 currently married<br>3 widow/widower<br>4 divorced/separated<br><br>For children, use 1 – never married	**HL7.**<br>What is your religion?<br><br>1 Roman Catholic<br>2 Protestant<br>3 other Christian<br>4 Muslim<br>5 Traditionalist<br>6 No Religion<br>97 Other (specify)	**HL8.**<br>What is your ethnic group?<br><br>01 Embu<br>02 Kalenjin<br>03 Kamba<br>04 Kikuyu<br>05 Kisii<br>06 Luhya<br>07 Luo<br>08 Masai<br>09 Meru<br>10 Mijikenda/Swahili<br>11 Somali<br>12 Taita/Taveta<br>13 Mixed<br>97 Other (Specify)	**HL9.**<br>What language does (name) speak?<br><br>Probe: Does (name) speak any other languages?<br><br>1 English<br>2 Swahili<br>97 Other (specify)

**ID**	**FIRST**	**LAST**	**RELATION**	**GENDER**	**MONTH**	**YEAR**	**AGE**	**MARITAL STATUS**	**RELIGION**	**OTHER**	**ETHNICITY**	**OTHER**	**LANGUAGE**	**OTHER**
01														
02														
03														
04														
05														
06														
07														
08														
09														
10														
11														
12														
13														
14														
15														

** Codes for **HL3**:<br>Relationship to head of household:*	01 HEAD<br>02 SPOUSE / PARTNER<br>03 SON / DAUGHTER<br>04 SON-IN-LAW / DAUGHTER-IN-LAW	05 GRANDCHILD<br>06 PARENT<br>07 PARENT-IN-LAW<br>08 BROTHER / SISTER	09 BROTHER-IN-LAW / SISTER-IN-LAW<br>10 UNCLE/AUNT<br>11 NIECE / NEPHEW<br>12 OTHER RELATIVE	13 ADOPTED / FOSTER / STEPCHILD<br>14 SERVANT (LIVE-IN)<br>96 OTHER (NOT RELATED)<br>98 DK

PANEL 1. HOUSEHOLD DEMOGRAPHY AND INFRASTRUCTURE

1.2. Education, Employment, and Migration

In this section, I would like to ask a few questions about education, employment, and migration history of each member in the household.

**HL0.**<br>*ID*	**HL10.**<br>What is the highest school grade completed by (name) so far?<br><br>00 Nursery<br>01 Std1<br>02 Std2<br>03 Std3<br>04 Std4<br>05 Std5<br>06 Std6<br>07 Std7<br>08 Std8<br>11 Form1<br>12 Form2<br>13 Form3<br>14 Form4<br>15 Form5<br>16 Form6<br>98 Don't know	**HL11.**<br>What kind of employment is (name) having?<br><br>01 paid employee<br>02 casual labour<br>03 household owns farm<br>04 livestock keeping<br>05 own business<br>06 employer<br>07 unpaid family worker/ housewife<br>08 apprentice<br>97 other (specify)<br>98 Don't know<br>99 Not applicable		**HL12.**<br>Was (name) born in this location?<br><br>1 yes – HL13<br>2 no – HL12a-d	**HL12a.**<br>Where was (name) born?<br><br>*Specific location, e.g., name of village, city, country, etc.*	**HL12b.**<br>[FOR ADULTS ONLY] How many years has (name) been living in this location?<br><br>*If not known, write 98*	**HL12c.**<br>[FOR ADULTS ONLY] Where did (name) live before moving into this location?<br><br>*If not known, write 98*	**HL12d.**<br>What was (name)'s main reason for moving into this location?<br><br>01 marriage/partnership<br>02 separation/divorce<br>03 join family<br>04 better economic opportunity<br>05 employment/transfer<br>06 better education<br>07 better health facilities<br>08 drought<br>09 seasonal<br>10 better infrastructure<br>11 death of caregivers/fostered/adopted<br>12 war/armed conflict<br>97 other (specify)<br>98 Don't know<br>99 Not applicable	
**ID**	**CODE**	**CODE**	**OTHER**	**CODE**	**SPECIFY**	**YEARS OF STAY**	**PREVIOUS PLACE**	**CODE**	**OTHER**
01									
02									
03									
04									
05									
06									
07									
08									
09									
10									
11									
12									
13									
14									
15									

PANEL 1. HOUSEHOLD DEMOGRAPHY AND IFRASTRUCTURE

1.3. Parents, Caregiver, and Child Education (only for children under 18)

In this section, I am going to ask some questions about the children under age 18 in the household. I need to talk to the parent or the main caregiver for each child.

**HL0.**<br>*ID*	**HL13.**<br>Is (***name***)'s natural mother alive?<br><br>1 yes<br>2 no<br>98 Don't know<br><br>2/98 -> HL14	**HL13a.**<br>Does (***name***)'s natural mother live in this household?<br><br>1 yes<br>-> write ID<br>2 no		**HL14.**<br>Is (***name***)'s natural father alive?<br><br>1 yes<br>2 no<br>98 Don't Know<br><br>2/98 -> HL15	**HL14a.**<br>Does (***name***)'s natural father live in this household?<br><br>1 yes<br>-> write ID<br>2 no		**HL15.**<br>Who is the main person who cares for this child?<br><br>99 child's mother<br><br>Otherwise, write the ID	**HL16.**<br>[FOR CHILD UNDER 2] Is (name) attending day-care or is taken care by anyone besides the parents on a regular basis?<br><br>01 daycare<br>02 grandparents<br>03 older siblings<br>04 relatives<br>05 neighbors<br>06 other (specify)<br><br>99 Not Applicable	**HL17.**<br>Is (name) currently enrolled in school?<br><br>1 yes<br>2 no<br><br>1->HL17b & HL17c<br>2->HL17a	**HL17a.**<br>What is the main reason (name) is not currently enrolled in school?<br><br>01 Sick<br>02 Sickness of family member<br>03 Working for family<br>04 Paid work outside household<br>05 House work<br>06 Away from home<br>07 Does not wish to attend<br>08 Cannot afford fees<br>09 Cannot afford uniform/supplies<br>10 School too far away/ no transportation<br>11 School full<br>12 School poor quality<br>13 Parent forbade it<br>14 Conflict with religious beliefs<br>15 Too old/finished school<br>16 Too young<br>97 Other (specify)	**HL17b.**<br>What is the level of school (name) is enrolled in?<br><br>00 Nursery<br>01 Std1<br>02 Std2<br>03 Std3<br>04 Std4<br>05 Std5<br>06 Std6<br>07 Std7<br>08 Std8<br>11 Form1<br>12 Form2<br>13 Form3<br>14 Form4<br>15 Form5<br>16 Form6<br>98 Don't Know	**HL17c.**<br>What is the type of school (name) is enrolled in?<br><br>1 Public<br>2 Private – church<br>3 Private – mosque<br>4 Private – other<br>5 Community<br>97 other (specify)	**HL18.**<br>Has (name) ever attended any early childhood education program?<br><br>1 yes<br>2 no<br>98 Don't Know	**HL19.**<br>Does (***name***) have a birth certificate (piece of paper proving birth registration)<br><br>1 yes<br>2 no<br>98 Don't Know<br><br>1->HL20	**HL19a.**<br>If not, why not?<br><br>01 too expensive<br>02 not necessary<br>03 do not know where to get one<br>04 place to get one is too far<br>05 do not have necessary documents<br>06 lost<br>07 burned<br>97 other (specify)	
**ID**	**CODE**	**CODE**	**ID**	**CODE**	**CODE**	**ID**	**CODE**	**CODE**	**CODE**	**CODE**	**CODE**	**CODE**	**CODE**	**CODE**	**CODE**	**SPECIFY**
01																
02																
03																
04																
05																
06																
07																
08																
09																
10																
11																
12																
13																
14																
15																

PANEL 1. HOUSEHOLD DEMOGRAPHY AND INFRASTRUCTURE		
**1.4. Household Assets and Infrastructure**<br>In this section, I would like to ask some questions about your household possessions, family income, and access to water and sanitation.		
**HL20.**<br>What is the main primary type of appliance used for cooking?	01 = Traditional stone fire<br>02 = Improved traditional stone fire<br>03 = Ordinary Jiko<br>04 = Improved Jiko<br>05 = Kerosene stove<br>06 = Gas cooker<br>07 = Electric cooker<br>97 = Other (specify) ___________________________________	
**HL21.**<br>Does your household own any of the following items?	01. Bicycle<br>02. Motorcycle<br>03. Radio<br>04. Telephone / mobile<br>05. Refrigerator<br>06. Fan<br>07. Bucket / Basin<br>08. Wooden Stool<br>09. Bed<br>10. Bed Sheets<br>11. Blankets<br>12. Mosquito Net<br>13. Table<br>14. Chair<br>15. Computer/tablets<br>16. Access to internet at home<br>17. Television	
**HL22.**<br>What kind of toilet facility does your household use?	01 = None<br>02 = Flush to sewer<br>03 = Flush to septic tank<br>04 = Pan/bucket<br>05 = Pit latrine with ground level cover over latrine<br>06 = Uncovered pit latrine<br>07 = Ventilation-improved pit latrine<br>97 = Other (specify) _________________________________	
**HL23.**<br>What is the main source of drinking water during the dry season?	01 = Piped into dwelling or compound<br>02 = Public outdoor tap or borehole with pump<br>03 = Protected well, rainwater or spring<br>04 = Unprotected well, rainwater, spring<br>05 = River, lake, pond<br>06 = Mobile vendor<br>97 = Other (specify) ____________________________________	
**HL24.**<br>What are the main sources of income for your household?	01 Salaried employment<br>02 casual labour<br>03 self employment – agriculture<br>04 self employment – non-agriculture<br>05 property / land rental /interest revenue<br>06 pension<br>07 gifts in cash and kind (relatives/friends)<br>08 transfers from government (cash transfers / food aid etc)<br>09 other private transfers (church/NGOs, etc)<br>97 other (specify) ____________________________________	a) Main (first) source:_______<br>b) Second source (if any):____<br>c) Third source (if any):______<br>99 = Not Applicable

PANEL 2. MATERNAL AND CHILD HEALTH

In this and the following panels, I would like to speak to the mother of a child who is under 2 years of age in this household.

2.1. Birth History and Antenatal Care

In this section, I would like to ask some questions about your birth history and what kind of antenatal care you have received during your pregnancy with (name).

**MH1.** How old were you when you were first pregnant?	Before I was 18 years old....1<br>After I was 18 years old....2
**MH2.** How many live births have you had?	
**MH3.** Was there a live birth in the last 2 years?	Yes....1<br>If yes, name:______________________<br>No....2<br>If no, go to 2.2
**MH4.** Did you see anyone for antenatal care during your pregnancy with (name)? Whom did you see?	Yes, I see a doctor/nurse/midwife....1<br>Yes, I see a traditional birth attendant....2<br>Yes, I see a community health worker....3<br>No one....4<br>If no one, go to MH8
**MH5.** Did you receive the antenatal care in Kawangware?	Yes....1<br>No....2<br>If no, specify where:___________________________
**MH6.** When was the first time you saw someone for antenatal care?	Before 3 months pregnant....1<br>After 3 months pregnant....2<br>Don't remember....98
**MH7**. How many times did you receive antenatal care during this pregnancy?<br>*Probe: Was it one, two or three times, or was it more than four times?*	1 to 3 visits....1<br>4 or more visits....2<br>Don't remember....98
**MH8**. Who assisted with the delivery of (***name***)?	Doctor....1<br>Nurse / Midwife....2<br>Traditional Birth Attendant....3<br>Community Health Worker....4<br>Relative / Friend....5<br>No One....6
**MH9**. Where did you give birth to (***name***)?	Home....1<br>Hospital....2<br>Health clinic....3<br>Health post....4<br>Other (specify)....97
**MH10.** Did you give birth to *(name)* in Kawangware?	Yes....1<br>No....2<br>If no, specify where:___________________________

2.2. Immunization

Immunization is very important for your child's health. In this section, I would like to learn about what vaccination your child has got.

**IM1**. Do you have a health card (e.g., vaccination card, growth monitoring card)?	YES....1<br>NO....2<br>*IF "NO", GO TO 2.3*

**IM2**. Record each vaccination on the health card.<br>1 if the vaccination is present on the card<br>2 if the vaccination is not present on the card		YES	NO
BCG	BCG	1	2
HepB (at birth)	HepB0	1	2
Polio (OPV) (at birth)	OPV0	1	2
Polio (OPV) 1	OPV1	1	2
Polio (OPV) 2	OPV2	1	2
Polio (OPV) 3	OPV3	1	2
Polio (IPV)	IPV	1	2
Pentavalent (DPTHibHepB) 1	Penta1	1	2
Pentavalent (DPTHibHepB) 2	Penta2	1	2
Pentavalent (DPTHibHepB) 3	Penta3	1	2
Pneumococcal (Conjugate) 1	PCV1	1	2
Pneumococcal (Conjugate) 2	PCV2	1	2
Pneumococcal (Conjugate) 3	PCV3	1	2

Rotavirus 1	Rota1	1	2
Rotavirus 2	Rota2	1	2
Rotavirus 3	Rota3	1	2
MMR/MR 1	MMR/MR1	1	2
MMR/MR 2	MMR/MR2	1	2
Yellow Fever	YF	1	2

**2.3. Care of Illness**<br>In this section, I would like to talk you about how you take care of your child when he/she is sick.	
**CA0.** Do you have a medical document from a public or private health provider where (***name***)'s medical records are written down?<br>If yes, use information from the medical document to assist answering the following questions.	Yes .... 1<br>No 2
**CA1.** At any time in the **last two weeks**, has (name) had diarrhoea?	Yes .... 1<br>No .... 2<br>*If yes, go to CA#a to CA#d & go back to CA2*
**CA2.** At any time in the **last two weeks**, has (name) had an illness with a cough?	Yes .... 1<br>No .... 2<br>*If yes, go to CA#a to CA#d & go back to CA3*
**CA3.** At any time in the **last two weeks**, has (name) had fast, short, rapid breaths or difficulty breathing?	Yes .... 1<br>No .... 2<br>*If yes, go to CA#a to CA#d & go back to CA4*
**CA4.** At any time in the **last two weeks**, has (name) been ill with a fever?	Yes .... 1<br>No .... 2<br>*If yes, go to CA#a to CA#d*
The following questions are follow-up questions if any of the above illnesses are reported. Replace # with the number corresponding to the illness. For example, if diarrhoea is reported, the follow-up questions would be CA1a to CA1d.	
**CA#a.** Did you seek any advice or treatment for the illnesses from any source?	Yes .... 1<br>No .... 2<br>*If yes, go to CA#b1.*<br>*If no, go to CA#b2.*
**CA#b1.** Where did you seek advice or treatment?	Government hospital .... 1<br>Government health centre .... 2<br>Government health post .... 3<br>Community health worker .... 4<br>Other public medical (specify) .... 5<br>Mobile clinic .... 6<br>Mission/church/mosque hospital .... 7<br>Private hospital or clinic .... 8<br>Pharmacy/chemist .... 9<br>Traditional healer .... 10<br>Relative/friend .... 11<br>Other (specify) 97
**CA#b2.** If you did not seek treatment, why not?	Cannot spare the time .... 1<br>Cannot leave other children behind .... 2<br>Place too far .... 3<br>Place too expensive to get to .... 4<br>Don't think treatment is of benefit .... 5<br>Illness not serious .... 6<br>Cannot afford fee for treatment .... 7<br>Cannot afford fee for drugs 8

	Queue too long ... 9<br>Other (specify) ... 97
**CA#c.** At any time during the illness, was (name) given any medicine for the illness?	None... 1<br>Antibiotics... 2<br>Other medications (specify) ... 97<br>Don't know ... 98
**CA1c1. [Only for diarrhoea]** During the time (name) had diarrhoea, was (he/she) given a fluid made from a special packet called Oralite for Oral Rehydration Salt packet solution?	Yes ... 1<br>No ... 2<br>Don't know ... 98
**CA1c2. [Only for diarrhoea]** What else was given to treat the diarrhoea?	Nothing ... 1<br>Tablet or syrup ... 2<br>Injection ... 3<br>Intravenous ... 4<br>Home-made sugar/salt solution ... 5<br>Home/herbal/traditional remedies ... 6<br>Other (specify) ... 97
**CA#d.** Where did you get the medicine?	Government hospital ... 1<br>Government health centre ... 2<br>Government health post ... 3<br>Community health worker ... 4<br>Other public medical (specify) ... 5<br>Mobile clinic ... 6<br>Mission/church/mosque hospital ... 7<br>Private hospital or clinic ... 8<br>Pharmacy/chemist ... 9<br>Traditional healer ... 10<br>Relative/friend ... 11<br>Other (specify) ... 97<br>Don't know ... 98

PANEL 3. Nutrition and Nutrition Practices

In this panel, I would like to talk to you about how you feed your child.

**BD1**. Has (name) ever been breastfed?	Yes .... 1<br>No 2
BD2. How long after birth did you first put (name) to the breast?	Within one hour .... 1<br>Within 24 hours .... 2<br>More than 24 hours .... 3<br>Don't know 98
BD3. Was (name) given anything other than breastmilk in the first 3 days after birth?	Yes .... 1<br>No .... 2<br>Don't know 98
**BD3b**. If yes, what was given to drink other than breastmilk?	Milk (other than breast milk) .... 1<br>Plain water .... 2<br>Sugar or glucose water .... 3<br>Gripe water .... 4<br>Sugar-salt-water solution .... 5<br>Fruit juice .... 6<br>Infant formula .... 7<br>Tea/infusions/traditional herbal preparations .... 8<br>Honey .... 9<br>Prescribed medicine .... 10<br>Other (specify) 97
**BD4.** Is (name) still being breastfed?	Yes .... 1<br>No 2
**BD5.** How many months old was (name) when he/she was first fed formula, liquids such as water, or semi-soft foods?	

BD6. Now I would like to ask you about everything that (name) ate yesterday during the day or the night. Please include foods consumed outside of your home.

- Think about when (name) woke up yesterday. Did (he/she) eat anything at that time?
  If 'Yes' ask: Please tell me everything (name) ate at that time. Probe: Anything else?
  Record answers using the food groups below.
- What did (name) do after that? Did (he/she) eat anything at that time?
  Repeat this string of questions, recording in the food groups, until the respondent tells you that the child went to sleep until the next morning.

If respondent mentions mixed dishes, ask: What ingredients were in that (mixed dish)?

	Yes	No
a) Baby food	1	2
b) Grains, roots or tubers	1	2
c) Vegetables or fruits	1	2
d) Meat, fish, poultry or liver/organ meats	1	2
e) Dairy products (e.g., milk products)	1	2
f) Eggs	1	2
g) Beans, lentils, nuts or seeds	1	2
h) Other soli,d, semi-solid, or soft food	1	2
Specify other:		

PANEL 4. CAREGIVING PRACTICES					
In this panel, I would like to talk to you about what you and your family do with your children in daily life.					
EC1. How many children's books or picture books do you have for (*name*)?	None .... 1<br>Number of children's books___________________<br>10 or more books 10				
EC2. Does (he/she) have any of the following types of toys at home:					
	YES	NO			
a) Homemade toys, such as dolls, cars, or other toys made at home?	1	2			
b) Toys from a shop or manufactured toys?	1	2			
c) Household objects, such as bowls or pots, or objects found outside, such as sticks, rocks, animal shells or leaves?	1	2			
EC3. Sometimes adults taking care of children have to leave the house to go shopping, wash clothes, or for other reasons and have to leave young children. On how many days in the past week was (name):	Number of days				
a) Left alone for more than an hour?					
b) Left in the care of another child, that is, someone less than 10 years old, for more than an hour?					
EC4. In the past 3 days, did you or any household member engage in any of the following activities with (name):<br>If 'Yes', ask:<br>Who engaged in this activity with (name)?<br>Record all that apply.					
	Mother	Father	Siblings	Other	Nobody
a) Read books or looked at picture books with (name)?	1	2	3	4	5
b) Told stories to (name)?	1	2	3	4	5
c) Sang songs to or with (name), including lullabies?	1	2	3	4	5
d) Took (name) outside the home?	1	2	3	4	5
e) Played with (name)?	1	2	3	4	5
f) Named, counted, or drew things for or with (name)?	1	2	3	4	5
EC5. Adults use certain ways to teach children the right behavior or to address a behavior problem. I will read various methods that are used. Please tell me if you or any other adult in your household has used this method with *(name)* in the past month.					
	YES	NO			
a) Explained why (name)'s behavior was wrong, took away privileges, forbade something (name) liked or did not allow (him/her) to leave the house, or gave (him/her) something else to do.	1	2			
b) Shouted, yelled or screamed at (him/her) or called (him/her) dumb, lazy or another name like that.	1	2			
c) Shook, spanked, hit or slapped (him/her) on the bottom with bare hand, hit or slapped (him/her) on the hand, arm, or leg, or hit (him/her) on the bottom or elsewhere on the body with something like a belt, hairbrush, stick or other hard object	1	2			
d) Hit or slapped (him/her) on the face, head or ears, beat (him/her) up such as hitting (him/her) over and over as hard as one could.	1	2			
